# A qualitative assessment of the impact of a community-embedded intervention on beneficiaries' attitudes and beliefs about adolescent sexual reproductive health in Ebonyi State, Southeast, Nigeria

**DOI:** 10.1186/s12978-024-01738-9

**Published:** 2024-01-11

**Authors:** Chibuike Innocent Agu, Ifunanya Clara Agu, Chinyere Ojiugo Mbachu, Nkoli Ezumah, Obinna Onwujekwe

**Affiliations:** 1 Health Policy Research Group, University of Nigeria Nsukka, Enugu, Nigeria; 2 Department of Community Medicine, University of Nigeria Nsukka, Enugu, Nigeria; 3 Department of Health Administration and Management, University of Nigeria Nsukka, Enugu, Nigeria; 4 Department of Community Medicine, Alex-Ekwueme Federal University Teaching Hospital, Abakaliki, Ebonyi Nigeria

**Keywords:** Community-embedded intervention, Adolescent sexual and reproductive health, Attitudes, Beliefs, Norms, Behaviour change, Nigeria

## Abstract

**Background:**

Adolescents and their communities in Ebonyi State, Nigeria have poor attitudes and beliefs towards adolescent sexual and reproductive health (SRH). This paper reports on the effects of a community-embedded intervention that focused on creating positive changes in the attitudes and beliefs of adolescents and community members to enhance adolescents’ access to SRH information and services.

**Methods:**

This study adopted the Qualitative Impact Assessment approach to evaluate the changes in attitudes and beliefs about the SRH of adolescents from the perspectives of the beneficiaries of a community-embedded intervention namely, adolescents, parents, school teachers, and community leaders. The intervention was implemented in six local government areas in Ebonyi State, southeast Nigeria and the evaluation was undertaken four months after the implementation of the interventions commenced. Eighteen (18) interviews were conducted with 82 intervention beneficiaries including: (i) six in-depth interviews with school teachers; (ii) two sex-disaggregated FGDs with parents; (iii) two sex-disaggregated FGDs with community leaders; and (iv) eight sex-disaggregated FGDs with in school and out of school adolescents. A thematic analysis of data was performed with the aid of NVivo software, version 12.

**Results:**

The community-embedded intervention led to changes in individual attitudes and beliefs, as well as changes in community norms and values concerning adolescent SRH. Adolescents reported that following the community-embedded SRH intervention, they have become more comfortable discussing openly SRH issues with their peers, and they could more easily approach their parents and initiate SRH discussions. The parents of adolescents reported that following the intervention, they have become more willing to discuss sensitive SRH issues with adolescents, and frequently make out time to do so. It was also reported that parents no longer use euphemisms to describe sexual body parts, and community leaders now believe that it is all right to discuss SRH with adolescents. Hence, initiating or having SRH discussions with adolescents is no longer misconceived as encouraging sex, and menstruation in unmarried adolescents is no longer viewed as a sign of promiscuity. Respondents also highlighted changes in community norms of, (i) gendered parental communication of SRH matters, as both mothers and fathers have started discussing SRH issues with their adolescent boys and girls; and (ii) public shaming and discipline of pregnant teenage girls are on the decline.

**Conclusion:**

The community-based intervention had a positive impact on individual attitudes and beliefs, as well as community and societal values and norms about adolescent SRH. Interventions that take into account community norms and values regarding adolescent SRH should be prioritized to enable the achievement of the SRH-related target of SDG 3.

**Supplementary Information:**

The online version contains supplementary material available at 10.1186/s12978-024-01738-9.

## Plain language summary

Adolescents face significant sexual and reproductive health (SRH) challenges which makes it difficult for them to access and utilize SRH services as a result of negative community norms and values. These norms discourage discussions relating to sex and sexuality in Nigeria because sexuality matters are regarded as taboo for young people, and sex is regarded as sacred and the exclusive reserve of the married. This qualitative study explored the views of adolescents, parents, and community leaders on the impact of a community-based intervention on their attitudes and beliefs concerning SRH issues. Using focus group discussions, the community members described the impact of the intervention on the beliefs and attitudes of adolescents, parents/guardians, and the community. Following the SRH intervention, parents started to make out time to discuss SRH issues with adolescents. They became more approachable and willing to discuss sensitive SRH issues with adolescents. Both mothers and fathers started to take up the role of communicating SRH matter with their adolescent boys and girls. Adolescents also started initiating SRH discussions with parents and could openly discuss SRH issues with their peers. In the community, adolescent girls were no longer publicly shamed for engaging in pre-marital sex. There were changes in community leaders’ attitudes to teenage pregnancy and they no longer misconceive adolescent SRH issues. Considering the positive changes in individual attitudes, social values, and norms, there is a need for the community-embedded SRH intervention to be sustained and scaled up to other parts of the state by program managers.

**Supplementary Information:**

The online version contains supplementary material available at 10.1186/s12978-024-01738-9.

## Background

The need to address adolescent sexual and reproductive health (ASRH) matters is a top priority in the world [1]. Hence, the World Health Organization (WHO) included adolescents in its 2016–2030 global strategy for women and children, highlighting the unique health challenges of adolescents [2, 3]. Some of these challenges include; poor access to adequate SRH information and services, child marriage, risky sexual behaviours, unintended pregnancies, unsafe abortion, and maternal mortality among adolescents [4]. More recently, the WHO, the International Planned Parenthood Federation (IPPF), and other organizations strongly called for further investment in adolescent sexual and reproductive health (ASRH) programming [3, 5]. However, in developing countries, young people face numerous structural, cultural, and social contexts that affect their access to and use of SRH information and services [6].

In Nigeria, like in some other sub-Saharan African countries, young people also face significant challenges such as low awareness about sexual and reproductive health services (SRHS), and financial constraints [7]. These, and negative community norms and values make it difficult for them to access and utilize SRHS, despite national policies promoting these services for adolescents and young people [8]. Community norms include norms that unmarried boys and girls should abstain from sex until marriage, and that sex-related discussions should not be held with young people [9, 10]. These norms discourage discussions relating to sex and sexuality because sexuality matters are regarded as taboo for young people, and sex is regarded as sacred and the exclusive reserve of the married [10, 11]. Communication between parents and adolescents on sexual health and reproductive-related matters rarely occurs, and even when it does, it mostly consists of strict warnings that may not protect adolescents from making unhealthy sexual and reproductive health choices [10]. Community attitudes toward SRH stem from stigma around premarital sex [7]. Communities disapprove of adolescents’ having access to SRHS, and so young people often end up being discriminated against if they do attempt to access SRHS [7, 10].

However, the goal of ASRH interventions, whether behavioural or structural is to reduce the negative consequences of risky sexual behaviour and promote healthy sexual relationships and SRH well-being for young people [12]. Whereas behavioural interventions seek to address individual beliefs, attitudes, and knowledge, structural interventions mostly aim to tackle the broader societal/community beliefs and norms that drive adolescent SRH behaviours using existing structures in the community [12]. Changes in social values, attitudes, perceptions, beliefs, and norms are made possible through information provided in the course of such interventions, leading to behaviour change. It has been shown that social values, beliefs, and norms are important factors in the formation of sexual behaviours [13, 14]. Attitudes about risky sexual behaviour are related to sexual behaviour in adolescents and other populations [15].

A systematic review on high risky behaviors described the risky sexual behaviours of adolescents at the personal, family, peer, school, and community levels [13]. These include personal factors such as knowledge and sources of information about sexual health, family structure and relationships, peer influence, religious beliefs, and influence of cultural and social changes in the community [13, 16]. Peers and parents are critical and have been identified as stable determinants of sexual behaviour [17]. Positive parental relationships are associated with lower levels of risk-taking and a reduction in the likelihood of early initiation of sex among adolescents. They are also, important in the formation of romantic relationships [18]. However, in places, like Nigeria and other SSA countries, where adolescent-parent communication regarding sex is not encouraged [33], older adults and parents can act as a barrier to adolescents’ health-seeking SRH behaviors [19].

ASRH interventions that are focused in changing social norms at the community and individual levels, while ensuring supportive policies and access to good quality services, can bring about significant improvements in adolescent-parent communication, sexuality, and other SRH issues [12]. Thus, properly designed and well-implemented interventions can increase effective parent-adolescent communication about sexuality and thereby reducing adolescent sexual risk-taking. A number of these interventions have been implemented in different contexts to transform attitudes, beliefs, and norms related to SRH among adolescents and other community members [20, 21]. Additionally, evidence suggests that programs are more likely to be successful if they include such community-based components [12, 22].

Although, ASRH has been on the health and education agenda for many years, there is still considerable apathy towards implementation of intervention programs in many countries [1]. Interventions are often delivered in a low dosage with limited effects, and in low and middle-income countries, ineffective interventions and ineffective ways of delivering them continue to be widespread [23]. This paper provides new knowledge on how a community-based intervention can improve the attitudes and beliefs of individuals and community members about adolescent SRH in order to inform SRH policy formulations and implementations in Nigeria. It provides information on the effects of a community-embedded intervention on the attitudes and beliefs of individuals and community members. This information was generated by exploring the views of adolescents, parents, and community leaders on the impact of the intervention on their attitudes and beliefs concerning SRH issues. The findings from this study will be useful to health policy makers, planners, and implementers and provide evidence for effective ASRH policy formulation and implementation in the state in particular and the country as a whole.

## Methods

### Conceptual framework

The approach used to evaluate the impact of the community-embedded intervention was informed by the Qualitative Impact Assessment Protocol (QuIP). This is an impact evaluation approach that draws on Contribution Analysis, and has been applied to a variety of behaviours in various fields, including healthcare [24]. The approach places project beneficiaries’ voices at the centre of the evaluation, enabling them to share and feedback their experiences in an open, credible, and respectful way [24, 25]. QuIP attempts to reduce the bias associated with asking people directly about the effects of interventions intended to benefit them, by referring to the intervention being evaluated as little as possible during the interview process [26]. It also, uses open-ended, exploratory questions to collect a much broader range of information rather than specific project- or programme-based questions. Where possible, field researchers who are completely independent of the organisation responsible for the actions being evaluated are used for the evaluation [25, 26]. Researchers are trained to conduct exploratory interviews, but are deliberately not given information about the organisation being evaluated or the theory of change behind its actions.

### Study area

This study was undertaken in Ebonyi state, one of the five states in the southeast geo-political zone of Nigeria. It has a 5533 km^2^ estimated land area and more than 40% of its total population is below 15 years of age [27]. Ebonyi state has an estimated annual growth rate of 2.7% and its adolescent population is projected to double by the year 2050. The adult literacy rate in Ebonyi state was 68.1% in 2018, while there is 64.1% secondary school attendance rate among young people in the state. The state is inhabited mainly by people of Igbo extraction and their main language is Igbo [28]. It has been reported that the state has a high maternal mortality rate (602 per 100,000 population), and 39.7% of these mortalities occur among adolescent girls aged 15–19 years. Further description of the study area can be found in our previously published papers [10, 29–31].

### Study design and study population

This study was descriptive cross-sectional qualitative research that used in-depth interviews (IDI) and focus group discussions (FGD) data collection methods to evaluate the impact of a community-embedded intervention that aimed to improve access to SRH information and services to adolescents in urban and rural areas of Ebonyi state, Nigeria. The intervention was implemented in selected communities and schools in six local government areas in Ebonyi State, southeast Nigeria. The evaluation was undertaken four months after the implementation of the interventions commenced.

The study population were the beneficiaries of a community-embedded intervention namely, in-school and out-of-school adolescent boys and girls aged 13 to 18, parents and guardians of adolescents, school teachers, and community leaders. The gender differences of participants who were interviewed is showed in Table 1.

**Table 1:** Gender distribution of interview participants

Participants	Gender		Age group (in years)	Number of interviews
	Male	Female		
School teachers	2	4	31–49	6 in-depth interviews
Adolescents in communities	13	12	14–18	4 Focus group discussions
Adolescents in the intervention schools	13	14	15–18	4 Focus group discussions
Parents/ guardians of adolescents	6	6	28–45	2 Focus group discussions
Community leaders	6	6	27–62	2 Focus group discussions
Total	40	42	–	18 interviews

### The intervention

A multi-faceted participatory intervention was implemented. The components of the intervention were, (i) one-on-one advocacy visits to community leaders and gatekeepers; (ii) community campaigns to improve awareness and understanding of the sexual and reproductive health and rights of adolescents among community leaders, parents of adolescents, and out-of-school adolescents; (iii) training of school teachers and peer educators on the provision of comprehensive SRH information; (iv) establishment or reactivation of school health clubs; and (v) training and supportive supervision of formal and informal healthcare providers. The intervention strategies were co-produced with key stakeholders in the State and community [35], and implemented with boundary partners from the health, education, and information sectors. The detailed descriptions of these intervention strategies are provided in the Additional file 1 and can also be found in a recently published article [32].

### Data collection

Detailed information on attitudinal and belief changes was collected from 82 beneficiaries of the interventions. Participants were purposively selected based on their participation in school-based or community-based adolescent SRH intervention activities. Eighteen (18) interviews were conducted with intervention beneficiaries including, six in-depth interviews with school teachers; two sex-disaggregated focus group discussions (FGDs) with parents; two sex-disaggregated FGDs with community leaders; eight sex-disaggregated FGDs with in school and out of school adolescents. The sex-disaggregated interviews were conducted to enable active participation of individuals. The FGD sessions had an average of 6–7 participants.

The data were collected using FGD guides that were adapted for adolescents, community leaders, parents/guardians of adolescents. The interview guides were developed for this study by a team of qualitative research experts, and they were pre-tested in a contiguous state. Participants were informed of the objectives of the study and their roles and rights in the study. Written informed consent was obtained from each participant before the interviews or discussions**.**

The interviews were collected by experienced qualitative researchers for one month (14th October to 19th November 2021) until saturation was achieved. The FGDs were facilitated by a moderator, a note-taker, and a local guide/translator. All the interviews were audio recorded with the permission of the participants. The interviews and discussions were held in convenient venues for the participants. The FGDs were conducted in the participants’ preferred language (local dialect or English language).

### Data analysis

The recorded discussions were transcribed verbatim following each session by the note-takers. All FGDs that were conducted in the local dialect were translated into English concurrently with the transcriptions. The notes were used to assign proper labels to the transcripts, and to further enrich the transcripts with nuances and non-verbal cues that were observed during the KIIs and FGDs. Each transcript was read by the interviewer or moderator, and further edited for spelling and punctuation errors.

Thematic analysis of transcripts was performed using deductive-inductive approach. At the first instance, a generic coding framework was developed a priori by four researchers, based on the project evaluation objectives. In order to test the framework, four detailed transcripts (2 FGD, and 2 IDI) were selected and coded independently by four researchers. Table 2 highlights the codes and their descriptions in the framework.

**Table 2:** Thematic coding framework and their descriptions

Impact of the intervention: any reference on how any of the components of the intervention namely advocacy (radio/television programmes, panel discussions, advocacy visits), health information (training of teachers, peer educators, school club establishment and community awareness) and health service interventions (capacity building of healthcare providers and supportive supervision), positively influenced beneficiaries	
Impact on adolescents	Any reference on how the SRH intervention (advocacy, health information and health service interventions) positively influenced adolescents’ values, beliefs in terms of dispelling misconceptions on sexuality, FP
Impact on community gatekeepers and parents of adolescents	Any reference on how the SRH intervention positively influenced community (community leaders and parents of adolescents) values/norms/beliefs in terms of dispelling myths and misconceptions about SRH
Impact on healthcare providers	Any reference on how the intervention positively influenced the attitudes and opinions and values of healthcare providers in adolescent SRH service delivery
Impact on policy/decision makers	Any reference on the impact of the intervention on the attitudes and opinions and values of policy/decision makers in adolescent SRH service delivery

Discrepancies were resolved before all the (18) transcripts were then imported into NVivo software (version 12). Each transcript was coded by two independent researchers and these merged into one NVivo software. Following the coding, the word query output was generated for each code and thoroughly read to identify themes and sub-themes. A total of thirteen (13) sub-themes emerged across the four themes. Table 3 below shows the four themes and thirteen (13) sub themes that emerged across the codes.

**Table 3:** Emergent themes and sub-themes across the thematic coding framework

Emergent themes	Emergent sub-themes
1. Changes in the beliefs and attitudes of parents	• Parents making out time to discuss SRH issues with adolescents • Parents are more willing to discuss sensitive SRH Issues • Parents are more approachable to adolescents
2. Changes in the beliefs and attitudes of adolescents	• Adolescents now initiate SRH discussions with parents • Adolescents, now, openly discuss SRH issues with their peers
3. Changes in the attitudes and beliefs of community leaders	• Community leaders, now, believe it is right to discuss SRH with adolescents • Community leaders no longer misconceive adolescent SRH issues • Change in community leaders’ attitude to teenage pregnancy
4. Changes in community norms and values	• Adolescent girls are no longer publicly-shamed for engaging in pre-marital sex • Menstruation in unmarried adolescents no longer viewed as a sign of promiscuity • Community members no longer view SRH discussion with adolescents as ‘morally’ wrong • Euphemisms are no longer used to describe body parts • Both mothers and fathers are taking up the role of communicating SRH matters with their adolescent boys and girls

## Results

The results are structured according to the themes that emerged from each code in the framework (Table 2). These are in terms of changes in the belief and attitudes of parents, adolescents, community leaders as well as the community as a whole are reported.

### Changes in the beliefs and attitudes of parents

#### Parents making out time to discuss SRH issues with adolescents

Many of the parents admitted to not making out time to discuss SRH issues with their adolescents prior to the intervention. However, because they had participated in the community campaigns, they understood the importance (and benefits) of having these discussions with their adolescents and they began to create time to discuss SRH issues with their adolescents. A parent who expressed herself had this to say,

*“Yes, because in the past when I had not heard about this campaign, I never made out time to discuss with my adolescents about their SRH, but now with the campaign I now find it necessary to discuss such issues with children” (FGD with Female parent Iz, P5*)

#### Parents are more willing to discuss sensitive SRH Issues

Also, a good number of participants who were averse to holding SRH-related discussions with their adolescent children reported their willingness to discuss such matters with them after being exposed to the intervention.

*“It used to be a difficult thing for me to bring my children together to talk to them about sexual intercourse. I was thinking that telling them those things means that I am exposing them to sexual intercourse, but I have gotten the appropriate information about it, …they have known how they can protect themselves and how to stay healthy during menstruation.”* Female community leader Ik, P6)

*“Before you can’t just call and discuss with your child on certain issues not until after the campaign, we became disposed and freer to discuss sensitive issues with our children, it yielded fruits since that time”* (FGD with male community leader IZ, P4)

#### Parents more approachable to adolescents

Some of the participants who were difficult to be approached by adolescents (by being harsh or discouraging), now find it necessary to encourage adolescents who may wish to come to them to do so without undue hindrance. A community leader, who stated that the results of the intervention were positive shared his opinion thus,

*“Let me chip in; gone are days when we parents don't freely have discussion with our children. With this teaching, we have come to realize that having round table discussion with our children helps a lot, so it fits into my roles and responsibilities because it added values and updated our efforts hence the results are obvious and positive. It has rather facilitated our efforts towards our wards because it has reduced our harshness and increased parental relationship and interactions in knowing the true sexual and reproductive health rights of our adolescents in the community.”* –(FGD with male community leader Ik, P3).

### Changes in the beliefs and attitudes of adolescents

#### Adolescents now initiate SRH discussions with parents

It was also observed that prior to the intervention, adolescents experienced difficulty in initiating discussions on SRH needs with their parents. However, following the intervention, they now do so without any form of restriction.

A female parent who expressed her mind described the ability of adolescents to initiate discussions with their parents as one the strongest intervention benefit among adolescent population.

*“Yes, yes the thing is that prior to team visit, it was difficult for a child to discuss certain things with papa or mama easily but right after the training many of them are coming out knowing what to discuss with mama and papa and this is the strongest part of the benefits the children have gained.*” (FGD with female parent Iz, P1)

Also, a community leader who described the impact of the SRH community campaign as novel noted that the adolescents now approach parents for discussions when the need arises.

*“It removed shame from them, they [adolescents] were shy to approach and discuss with us but the campaign gave them leverage by exposing them to those they can discuss with and how to approach their parents when they feel like discussing these issues; a new dawn began in our lives.*” (FGD with male community leader Iz, P2)

#### Adolescents, now, openly discuss SRH issues with their peers

Some of the participants, especially adolescent girls, who believed that SRH issues should not be discussed in the open, perhaps due to fear of being stigmatized or embarrassed, noted that they are now emboldened to talk about such issues in the open when necessary. Others who used to feel shy discussing such matters, also reported that they no longer feel so. Thus, some of the participants now have flexible beliefs about sexuality.

#### Overcoming fear of stigmatization

An adolescent who made her opinion known during an FGD with adolescent girls stated that she had been equipped with SRH information and that she now feels bold talking about SRH matters even in the open.

*“I can now be bold to engage my mates in discussions about sexual and reproductive health issue, even in the open because I have been equipped here as a result of the intervention.”*

*“Before now I didn’t talk to people about SRH but now, I share my knowledge and experiences any time I see a group of people without being embarrassed”* (FGD, adolescent girls in Nw, P5)

#### Becoming more confident

*“There are great differences in the way I live. Before now, it was not easy for me to discuss SRH, I used to feel shy but since the training, I feel free to discuss it with my friends”* (FGD, adolescent girls in Nw, P3)

*“Before the campaign, I was shy to discuss such things amongst many people as we are gathered here but since the campaign I can freely talk about it.”* (FGD, adolescent girls in Nw, P6)

### Changes in the attitudes and beliefs of community leaders

#### Some community leaders, now, believe it is right to discuss SRH with adolescents

Some of the participants reported changes in their beliefs about discussing SRH with adolescents. Before the SRH intervention, many of them believed that it was wrong to discuss SRH issues with adolescents, or even to disclose SRH matters to them. Some others thought it was wrong to talk about it in the open. However, following the intervention, such beliefs gave way for positive ones as some of them reported that they now discuss or disclose such issues to adolescents.

*“Before now, I thought it was wrong to discuss SRH with adolescents but I have, now, known that it is important to start such discussion early as it has so much to change in their lives because we are in new age, I no longer see such discussion as something bad.”* (FGD with female community leader Ik, P1),

A participant who had a deeply entrenched belief (as a result of his upbringing) that SRH issues should not be disclosed to adolescents dropped that belief, following the intervention.

*“As a local farmer in the village it has affected my belief because before now I never knew that sexual and reproductive health issues should be disclosed with adolescents, I thought it should not be discussed or made known to them due to our own upbringing from childhood but the intervention has changed my perceptions about this hence I now discuss it freely with my children.”* – (FGD with male community leader Ik, P6).

#### Community leaders no longer misconceive adolescent SRH issues

Many of the participants disclosed that the intervention was instrumental to dispelling some misconceptions among them. Some of the misconceptions were that menstruation in girls between the ages of 10 to 19 means that the girl had sex, and that providing SRH services amounted to encouraging them to engage in sex.

*“On my own part before now I believed that for a girl to start menstruating at 10 to 19 years that the girl must have had sexual intercourse. But in the course of the training, I discovered that it is not true, so the training has cleared my eyes as I now know is a natural phenomenon which has no relationship with sexual intercourse or even the age of the girl.” (*FGD with m*ale, community leaders Ik, P3)*

#### Change in community attitude towards teenage pregnancy

Even though communities still abhor teenage pregnancy, the intervention changed the attitude of the communities towards unmarried teenage girls that become pregnant.

##### Now take care of pregnant adolescents

Generally, respondents described teenage pregnancy among unmarried adolescent as a concern. Before the community campaign, adolescent girls who got pregnant out of wedlock, were forced into a marriage with the males responsible for the pregnancy. Also, such pregnant adolescents would be maltreated and subjected to public ridicule, but the community campaign changed the attitude of the communities towards pregnant adolescents. These days, victims of teenage pregnancies are taken care of and are not maltreated or subjected to public ridicule.

*“It impacted our beliefs because before now, whenever a girl is pregnant, they will tie stone on her neck and force her to go to whoever that is responsible for the pregnancy but now, if any girl is pregnant, we will take her to the hospital and take proper care of her” – (*FGD with f*emale community leader Ab, P3)*

##### No longer force them into marriage

Before now, families insisted that their pregnant adolescent girl must marry anybody that is responsible for her pregnancy but now, it is no longer enforced.

*“If any girl happens to be pregnant, she is no longer forced into a marriage, she stays in her father’s house. After she delivers, she will wait for a man that will marry her”* – (FGD with f*emale community leader* Ab, P5)

##### Adolescent girls are no longer publicly shamed for engaging in pre-marital sex

Community leaders revealed that sexual relationships among unmarried adolescents are not culturally acceptable. Preceding the community intervention, adolescents who did not conform to the community rules of abstaining from premarital sex and became pregnant were publicly shamed and punished.

*“Before we have our control measures such that if you misbehave in the community especially if a girl had sexual intercourse with a boy in the way that does not conform with the set rules, culture and tradition of the community, such adolescents are usually disgraced in public by bonding [beating] and canning. We use the local handcuff and cane as corrective measures but since your awareness intervention such has reduced drastically”* **–(**FGD with *male community leader* Ab, R1)

### Changes in community norms and values

#### Menstruation in unmarried adolescents no longer viewed as a sign of promiscuity

It was established across parents and community leaders that some parents and community members believed that a girl can only start menstruating when she becomes sexually active. Hence, they believed that the adolescent has become promiscuous and this was seen as a crime. The parents of adolescents used to humiliate and shame them during their first menstruation These were mostly parents with non-formal education or mothers who had early marriage and consequently experienced their first menstruation after marriage. However, the SRH campaign provided both adolescents and their parents appropriate SRH information including anatomical changes in the body.

“The campaign has opened our eyes towards menstruation in adolescent girls as we were seeing it as an abomination and felt that any unmarried girl menstruating must have had sex, but now we have understood the true situation regarding menses, and so we long shame girls who menstruate before marriage”. (FGD with female community leader Ab, P2).

“It has changed some of our beliefs about menstruation in adolescents who are yet to marry. Before now, there used to be misconceptions especially about their first menstruation on whether they have started sleeping with men, but the community members know better” –(FGD with male community leader Ik, P3)“

In the past, whenever a young girl begins to menstruate, her parents will begin to see it as a crime, they will begin to use bad words on her, saying that she has defiled herself. Since after the teaching, things changed, we were told that there is need to make out time to teach our children about their sexual and reproductive health and rights” – (FGD with female community leader Ik, P4).

#### Community members no longer view SRH discussion with adolescents as ‘morally’ wrong

In the communities, discussions about SRH matters with young people were viewed as forbidden because individuals who engaged in such discussions were regarded as spoiling or corrupting such adolescents. Parents were not able to communicate effectively with their adolescent boys and girls due to such restrictive beliefs and values. However, the SRH campaign brought about changes as parents now freely discuss with their children on several topics such as; anatomical and physiological changes in the body during puberty, and matters relating to sexual health and experiences with opposite sex. Here are some supporting quotes from parents;

*“Before now, such discussion is “a no-go area” you can discuss other things with your children but for sexual health, you cannot mention those areas especially to a young person, people will look at you as a wild person but now, parents and children can freely talk. They can talk to their children about sexual and reproductive health. Parents and children can feely discuss about parts of their bodies and ask questions when there is need, the fear and shyness have been dealt with and discussions can go on” – (FGD with female parent Iz, P3).*

*“The campaign helped us, in the past parents cannot discuss parts of the body with their children, nobody can mention things like breast or buttocks instead they call it bombom but this campaign has made us to understand the need to call those parts of the bodies by their names.” – (FGD with Female community leader Ik, P2).*

*“It was very relevant because if the campaign did not take place, we would have been living in ignorance and ignorance kills. Some parents do shy away from telling their children the truth and they feel that it is not proper to talk about those things (sex-related matter) but now, they can freely talk and they have succeeded in correcting some things through information. The campaign exposed us to knowing the true situation of things and how things are supposed to be” – (FGD with Male parent Iz, P2*).

#### Euphemisms are no longer used to describe body parts

The community members reported that they now call body parts, especially the reproductive organs by their names. It was stated that before the intervention, it was their norm not to call any reproductive organ by their name, but they now feel free to do so during child-parent discussions. This helps them to express their opinions clearly and without any ambiguity during such discussions. Here are some supporting quotes from parents;

*“Not only that parents could not freely discuss sex-related topics with their children, but both male and female sex organs were not called by their names. We had mild ways of calling them without being embarrassed, but as a result of you people’s programme, we feel alright calling them by their names” (FGD with Male community leader Ab, P2)*

*“In this community, you can see an adult advising a girl child on matters concerning sex and he/she would not feel somehow to mention the names of the sex organs. This was not so before you came with the intervention.” (FGD with Male parent Iz, P3)*

#### Both mothers and fathers are taking up the role of communicating SRH matters with their adolescent boys and girls

Exploration of parent–child communication of SRH discussions revealed shifts in gendered roles of parents about parent–child communication of SRH discussions. Before the implementation of SRH intervention, parents and community leaders revealed that it was against their belief that a father should discuss SRH matter with their adolescent girls as this was believed to be the primary responsibility of mothers in the family. Mothers were described as those who are better positioned to understand their daughters than fathers. However, the implementation of SRH intervention strategies in the community brought about positive changes in their beliefs and perceptions of the pattern of parent–child communication. Parents now understand that both mother and father have roles to play in adolescent sexuality education, and can discuss SRH issues with any of their adolescent children.

*“At the earlier stage, it [SRH discussion with adolescents] didn’t fit into the type of lives we lived but, the teaching helped us to have balanced home where we can freely engage our children in sex-related discussions. Before, it was kicking against our cultural norms, because we never believed that a man can sit with his daughter to discuss her SRH. These responsibilities were kept specifically for mothers and they had limits to what they can say to their children. But with the intervention, both parents can freely talk to their children without any fear as it is longer frowned upon –(FGD with Male parent Iz, P1)*

*“It has a great effect in the communication between parents and children because parents have begun to see that it is their responsibilities to talk to their children and the adolescents have understood that it is not an abomination for a parent to invite any of his or her children for such discussions”–* (FGD with Male community leader Iz, P5).

*“I now know that my husband or I can discuss with any of our children, being a girl or a boy, on this SRH issue without shying away from it. This is possible because of the intervention. You know this local rural environment where many things are seemingly avoided, so we never talked freely with the adolescents*” – (FGD with Female parent Iz, P3)

## Discussion

The findings from the intervention show improvement in individual attitudes and community norms concerning adolescent sexual and reproductive health. Adolescents are now more confident to openly discuss SRH issues with their peers, while parents are more approachable and willing to discuss sensitive SRH issues with adolescents. The communities no longer misconceive menstruation in an unmarried adolescent, and their attitude towards teenage pregnancy has changed. The previous norm of maltreating adolescents with teen pregnancy by the communities is no longer practiced.

The finding that adolescents can now readily approach their parents, and sometimes even initiate discussions on SRH with parents is contrary to the situation prior to the implementation of the intervention when adolescents, did not feel comfortable discussing SRH matters with their parents because parents were not open-minded and easily misunderstood them [33]. This is an indication of increasing positive parental relationships in the communities, which are critical to many aspects of adolescent development [19]. The finding is comparable to the result from an evaluation study in the United States of America which reported a significant increase in the number of adolescents who approached their parents for discussions on different aspects of SRH, following a community-based SRH intervention [33]. The finding that adolescents are now more confident to engage their peers on SRH issues openly without fear of being stigmatized is similar to results from Northern Nigeria and South Africa [34, 35].

The fact that parents were now more willing to discuss SRH matters with their adolescent children and now make out time to have such discussions, is an improvement from the findings of an earlier study on parent-adolescent communication in the study area [10]. The result is important because, although parents often have difficulties in talking with their adolescents about SRH, they should be the primary educators of their children on such issues. Indeed, many studies support the position that the family is a primary source of information to adolescents on SRH matters [36, 37]. Parent-initiated communication should be the principal channel for conveying knowledge and values to adolescents, including information related to their sexual and reproductive health (SRH) [10].

Similarly, both mothers and fathers are taking up the role of communicating SRH matters with their adolescent boys and girls. Before the intervention, communication between parents and adolescents on SRH-related matters rarely occurred, and when it did, it was mostly with mothers. The norm was for mothers to talk to daughters, while fathers talked to sons [33], This is still the case in several sub-Saharan African countries where mothers continue to play a greater role in family sex education [38]. But following the SRH intervention, fathers and mothers in the study communities now feel free to talk to any of their adolescent children irrespective of their sex. This shift in the gendered roles of parents regarding parent–child communication of SRH discussions is likely to benefit adolescents who stand a better chance of getting balanced SRH information from both parents.

Our study also shows that adolescent or teen pregnancy is no longer viewed as immoral and an offense to be punished because there has been an improvement in the attitude of the communities to teen pregnancy. Community members now rally round an adolescent with pregnancy and offer support to her. Such persons are no longer seen as villains, but as victims to be protected and helped. Members of the communities now take care of them and assist them to seek appropriate SRH services from health facilities. The community norm of forcing adolescents into early marriage for being pregnant has also been stopped; instead, such pregnant teens now receive support and acceptance from their families and community members. Such a shift in attitude and norm might be useful for better understanding the causes of teen pregnancy while also offering an opportunity to address the problem of teen pregnancy. Indeed, the problem of teenage pregnancy must be solved within the context of the individual, family, and the community [10, 28]. Therefore, this line of action should be encouraged and strengthened while more efforts are made to promote contraceptive use among sexually active adolescents to achieve teen pregnancy prevention.

The findings showed that the improved understanding of what menstruation is all about community members no longer perceive menstruation in unmarried adolescents in a negative light. Previous studies highlighted the need to equip parents with appropriate knowledge to educate adolescents regarding their reproductive health as many adolescent girls who held false conceptions regarding menstruation were informed by their mothers [39, 40].

The use of qualitative research approach in this study limits the ability to generalize findings, and there could be social desirability and information bias due to the sensitive nature of the topic. However, triangulation using both IDI and FGD data, and engaging different population groups to gain a holistic understanding of the impact of the intervention was undertaken. The findings of the study were also presented to the interviewees in the process of participant validation (member-checking) and review to ensure credibility.

## Conclusion

All in all, the community-based intervention had a significant positive influence on the attitude and beliefs of individuals and community members about adolescent SRH. Adolescents can now readily approach their parents to discuss SRH issues. Both mothers and fathers are taking up the role of communicating SRH matters with their adolescent boys and girls. Community members no longer use euphemisms to describe body parts, they care for pregnant adolescents and no longer force them into early marriage. These findings also highlight the importance of engaging different key stakeholders in the community when coproducing and implementing the designed intervention strategies.

The ASRH programs that take into account community norms and values regarding adolescent sexual and reproductive health should be a priority if the sexual and reproductive health-related sustainable development goals (SDGs) are to be realized. Considering the positive changes in certain negative social values and norms, there is the need for the community-embedded SRH intervention to be sustained and scaled up to other parts of the state by program managers.

### Supplementary Information

**Additional file 1.** A detailed description of the SRH intervention.

## Data Availability

Additional data from the research project would be made available by the corresponding author on reasonable request.

## Abbreviations

SDGs: Sustainable development goals
FGDs: Focus group discussions
SRH: Sexual and reproductive health
QuIP: Qualitative impact assessment protocol
ASRH: Adolescent Sexual and Reproductive Health
IPPF: International Planned Parenthood Federation
WHO: World Health Organization
