# Supplementary material for: A qualitative assessment of the impact of a community-embedded intervention on beneficiaries' attitudes and beliefs about adolescent sexual reproductive health in Ebonyi State, Southeast, Nigeria
Source: Reprod Health. 2024 Jan 11;21:5. doi: 10.1186/s12978-024-01738-9 (PMC10782563; doi:10.1186/s12978-024-01738-9)
Supplement: Supplementary file 1 — Additional file 1. A detailed description of the SRH intervention. [file 12978_2024_1738_MOESM1_ESM.docx]

**Additional material 1 - A detailed description of the SRH intervention**

***One-on-one advocacy visits to traditional rulers, community gatekeepers, and a consultative forum with the council of traditional rulers***

As one of the strategies for improving access to SRH services for adolescents, advocacy visits to the six communities that were purposively selected in Ebonyi State, Nigeria for the study were implemented from October to December 2019. The community visit schedule was developed and shared with the ASRH advocacy implementation team. Each community was visited once by a team of academic researchers, adolescent-friendly health service providers, adolescent health focal persons, a representative of civil society organizations, and a State government media representative. During each visit, community leaders were addressed by two selected team members and the adolescent health focal person in each of the communities. One of the speakers emphasized the need for adolescent SRH services and strategies that have been designed to deliver these services to them, while the other speaker highlighted the contributions of community leaders in promoting access to these health services. The adolescent health focal person in each of the communities translated some of the discussions that were unclear to the traditional leaders, community, and religious leaders. Most persons who honored the invitation were traditional leaders, women leaders, men leaders, different religious leaders, community leaders, youth leaders, and social mobilization officers. During these visits, all the community leaders recognized that adolescents in their communities had sexual and reproductive health needs which ought to be addressed. They expressed a willingness to mobilize adolescents and other community members to utilize and benefit from the youth-friendly health services.

***Community awareness campaigns on adolescent sexual and reproductive health:***

The community awareness campaigns comprised group discussions with adolescents, parents and guardians of adolescents, and community leaders. This strategy leveraged on the existing capacity for ASRH in the communities (including trained school teachers, peer educators, and health workers) to deliver information to smaller groups of the target audiences on adolescent sexual and reproductive health. Before the small group information campaigns, information and education communication (IEC) materials (handbills and posters) were designed to provide quick information on adolescent sexuality, parent-child communication, and the prevention of COVID-19. These IEC materials were pasted by the community resource persons in strategic places such as community squares, town halls, market squares, churches, schools, PMV (chemist) stores, primary health centers, and youth-friendly centers.

The small group ASRH information campaigns commenced on the 23rd of July 2020 in two LGAs (Abakaliki and Izzi) with the plan to test the strategy, learn lessons and then scale up to the remaining LGAs. Drawing on the experiences gained and lessons learned from piloting the community campaigns in two communities, the intervention components were scaled up to the remaining four communities in Ezza south, Ikwo, Afikpo south, and Ohaozara LGAs.

The target audiences were adolescent boys and girls, parents and guardians of adolescents, and community leaders. In each community/LGA, five interactive campaign sessions were held with a maximum of 20 participants. Three (3) separate sessions were organized for adolescent boys and girls, one (1) session was organized for parents and guardians of adolescents, and another session was organized for community leaders/influencers (including religious leaders, traditional leaders, village heads, youth leaders, women leaders, men leaders and representatives of community vigilante). The sessions with adolescents and adults were facilitated by boundary partners comprising trained school teachers, peer educators, and adolescent health focal officers of the LGA, researchers from HPRG provided technical support to the boundary partners. Topics covered with adults include prevention and reporting of rape, sexual exploitation and abuse of adolescents; effective parent-child communication of SRH matters; and prevention and reporting of COVID-19 infection. Topics discussed with adolescents included: adolescent sexuality, SRH rights of adolescents, sexual abstinence, contraception and prevention of unwanted pregnancy, life skills for preventing rape, sexual exploitation and abuse, reporting of rape, sources of SRH information and services, and prevention and reporting of COVID-19 infection. The last session of the community campaigns was held on the 8th of October 2020.

***Training of teachers and peer educators on the provision of SRH information to adolescents***

Two- training workshops (one for teachers and the other for peer educators) were organized to build the capacity of secondary school teachers and peer health educators on the provision of comprehensive sexual and reproductive health (SRH) information to secondary school students in Ebonyi state. Six public secondary schools used for the baseline study were purposively selected for the training workshop. The school principal, a biology teacher, a health education teacher, a guidance counselor (G&C), and three senior secondary students were purposively selected from each school and invited to the workshop. Students trained as peer educators were selected by the principals of the secondary schools using a set of criteria given to them by the researchers.

The workshops were facilitated by community health physicians with adolescent health expertise and program boundary partners (guidance counselor Ebonyi state university; school health coordinator in Ebonyi state ministry of health –SMOH; adolescent focal manager family-life and HIV/AIDS education in Ebonyi state ministry of education -SMOE; state reproductive health and family planning focal manager SMOH; state adolescent health focal manager MOH; director administration and adolescent focal manager family-life and HIV/AIDS education in Ebonyi state universal basic education board -UBEB) who took part in designing the implementation strategy and tools. On the first day of the training, participants’ knowledge of SRH was evaluated using a test questionnaire (pretest). The training was conducted using a training manual specifically developed for the project intervention strategy. The manual consisted of eight modules on various aspects of adolescent SRH, as well as, on principles and practice of counseling, which specifically targeted the teachers. On the last day, both the teachers and students had a combined session on the processes of establishing school health clubs. Their knowledge of adolescent SRH was, evaluated using the same questionnaire (post-test), and results showed significant improvements in their knowledge of SRH. In the end, all participants received a manual and each school was given a protocol for establishing school health clubs.

***Formal inauguration of school health clubs and induction of club members:***

There was the inauguration and supervision of adolescent sexual and reproductive health (ASRH) clubs in secondary schools between March 2021 and June 2022. Following the training of school teachers and peer educators, interested students were recruited into school health clubs to promote access to comprehensive SRH information for adolescents in the six intervention secondary schools. The formal inauguration of the ASRH clubs and the induction of members of the club was implemented by the research team in collaboration with boundary partners from the SMOH, State Ministry of Information, SMOE, and UBEB. Each secondary school was visited by a team of researchers and boundary partners for the inauguration event. The following materials were provided to the ASRH clubs in each school: (i) club register; (ii) branded face caps; (iii) branded polo shirts; (iv) branded note pads and pens; (v) manuals for training adolescents on SRHR; and (vi) fliers and posters on the SRHR of adolescents. A billboard of the ASRH club was also mounted at the entrance of each school to brand it as an ASRH-promoting school.

***Training of health workers on the provision of comprehensive sexual and reproductive services for adolescents***

A total of 29 state-level healthcare workers were trained by public health physicians with some expertise in adolescent health. The purpose of the training workshop was to raise a critical mass of trainers who are competent and skilled to train other health workers in the provision of adolescent-friendly SRH services, and who would be available in Ebonyi state, beyond the life of the project, to continue providing this capacity building intervention. The training was conducted in different stages. After the training of state-level health workers, a workshop was organized for the officers in charge (OIC) of primary healthcare facility (PHC) and with close supervision 81 of them were trained by the state-level trainers. Then, two different workshops were organized for the community health workers (CHWs) and the patent medicine vendors (PMVs). In total, 80 voluntary CHWs, and 80 PMVs purposively selected from the six LGAs were trained to provide youth-friendly health services, including counseling and referral services. The training of trainers manual was specifically developed for state-level trainers and OICs of PHCs while two different manuals were designed for the PMVs and CHWs. This is to ensure that the manuals are simple and easy for each group to understand. The manuals consist of eight modules namely: i) introduction to adolescence and adolescent health; ii) sexuality and sexual behaviors; iii) sexually transmitted infections; iv) principles and practice of counseling; v) pregnancy and prevention of pregnancy; vi) counseling practices on selected health issues of adolescents (including values clarification); vii) optimal adolescent and youth-friendly services; and viii) record-keeping and health information systems.
